# A Low-Cost MEMS Missile-Borne Compound Rotation Modulation Scheme

**DOI:** 10.3390/s21144910

**Published:** 2021-07-19

**Authors:** Xiaoqiao Yuan, Jie Li, Xi Zhang, Kaiqiang Feng, Xiaokai Wei, Debiao Zhang, Jing Mi

**Affiliations:** 1 National Key Laboratory for Electronic Measurement Technology, North University of China, Taiyuan 030051, China; yxq978513@163.com (X.Y.); b1506011@st.nuc.edu.cn (K.F.); b1806023@st.nuc.edu.cn (X.W.); zhangdebiao@aliyun.com (D.Z.); s1806038@st.nuc.edu.cn (J.M.); 2 School of Electrical Control Engineering, North University of China, Taiyuan 030051, China; zhangxi@nuc.edu.cn

**Keywords:** rotation modulation, highly dynamic environment, MEMS, error modulation

## Abstract

Rotation modulation (RM) has been widely used in navigation systems to significantly improve the navigation accuracy of inertial navigation systems (INSs). However, the traditional single-axis rotation modulation cannot achieve the modulation of all the constant errors in the three directions; thus, it is not suitable for application in highly dynamic environments due to requirements for high precision in missiles. Aiming at the problems of error accumulation and divergence in the direction of rotation axis existing in the traditional single-axis rotation modulation, a novel rotation scheme is proposed. Firstly, the error propagation principle of the new rotation modulation scheme is analyzed. Secondly, the condition of realizing the error modulation with constant error is discussed. Finally, the original rotation modulation navigation algorithm is optimized for the new rotation modulation scheme. The experiment and simulation results show that the new rotation scheme can effectively modulate the error divergence of roll angle and improve the accuracy of roll angle by two orders of magnitude.

## 1. Introduction

As inertial navigation system (INS) has advantages of high autonomy, low cost, and small size, and it is widely used in a variety of ships, aerospace, vehicles, aircraft. However, the measurement errors of the inertial devices themselves introduced into the navigation solution cause the errors to accumulate over time [1,2,3,4]. Especially in the practical application of missile environment, long-term storage will lead to constant error drift, and the special application conditions determine that the IMU, which is mounted on the missile, cannot timely and accurately calibrate in advance [5]. RM technique is used to modulate the constant and slowly changing drift into sine/cosine form by periodically rotating one or more axes of the inertial navigation system [6]. Therefore, the introduction of RM can significantly improve navigation accuracy while meeting the high autonomous requirements of the missile environment [7]. With the development of research in recent years, the application of rotation modulation in high precision inertial navigation systems such as FOG has been relatively well established. However, research on navigation systems based on MEMS sensors is still in its infancy [8]. As MEMS has the characteristics of low cost and small volume, further research on the application of rotational modulation technology in MEMS navigation systems is of great significance for reducing the cost of high precision systems and improving the navigation accuracy of conventional missile-borne environments [9].

Rotary strapdown inertial navigation system based on its axis of rotation number can be divided into the single-shaft rotary strapdown inertial navigation system, the biaxial rotating strapdown inertial navigation system, and triaxial rotary strapdown inertial navigation system [8,10,11]. For these systems, a variety of inversion schemes can be designed. In the literature, gyroscopic measurements are used to control the rotation of the IMU, the angular motion of the modulation axis is isolated, and the measurement accuracy of single-axis rotation modulation is improved. At the same time, the strapdown algorithm is used to obtain the navigation information of various rotational modulation schemes [12,13]. Dual-axis rotation modulation can effectively suppress the constant error of three axes [14]. Many rotation schemes are designed for the system by periodically rotating alternately about the two axes, with positive and negative rotation about each axis, and with the motions of each axis being symmetric in space and time, in each rotation period [15]. Different rotation schemes are designed to minimize systematic errors. Various rotation modulation schemes designed for fiber optic gyroscope (FOG) have strong reference significance for MEMS inertial navigation [16,17]. However, compared with the fiber optic gyroscope, the MEMS sensor has a larger constant error and larger noise, and the error component is more complex [18]. The traditional rotary modulation method of fiber optic gyroscope directly applied to MEMS inertial system cannot adapt to a more complex application environment. This problem is an urgent problem and challenge to improve the accuracy of MEMS-based rotary modulation systems.

To solve this problem, the research based on MEMS rotary modulation system includes two aspects: rotation scheme and error analysis [19,20]. Different forms of rotation schemes are compared, according to which a single-axis continuous reciprocating rotation scheme shows better performance [21]. In order to further compensate for the error, the error characteristics of the single-axis rotation modulation system are analyzed, and the error modeling is carried out [22]. The error models of single-axis unidirectional continuous rotation, single-axis reciprocating rotation, and single-axis multiposition reciprocating rotation are analyzed and compared. The single-axis MEMS rotation modulation error is analyzed and compensated [23]. However, special requirements in highly dynamic environments are not considered [24]. In a highly dynamic environment, a novel rotation scheme is designed to compensate for the modulation angular rate instability in the high spin state [25]. After isolating the angular velocity of the projectile body, the accuracy of the inertial navigation system under a high rotation state is improved by uniaxial rotation modulation [26]. In terms of data processing, the existing particle filter, Rao–Blackwellised particle filter and extended Kalman filter (EKF) are not suitable for fast error compensation under highly dynamic conditions due to their long estimation time and complex calculation [27,28,29]. In highly dynamic environments, much research has been conducted on rotation modulation schemes and error compensation. However, the error of the modulation axis direction cannot be modulated by the traditional single-axis rotation modulation [30,31], and more importantly, the constant error of MEMS gyro is large, which results in the continuous accumulation of the error of the modulation axis, limiting the improvement of the navigation precision of the system [32,33]. This is the bottleneck that should be overcome to improve the accuracy of the system.

Since ammunition in highly dynamic environments is of small volume, low cost, high precision demand [34], this paper proposes a new compound rotating modulation scheme based on MEMS inertial sensor. The new solution preserves the small size of the traditional missile-borne single-axis rotating modulated MEMS inertial sensor and the simple control of the rotation scheme and reasonable modulation period, which is suitable for a high updating rate of data in a highly dynamic environment. Compared with the single-axis rotation modulation system, the new system inhibits the error accumulation and divergence in the axis of rotation modulation, and the attitude error of roll angle is reduced. Pitch angle attitude error is reduced. The yaw angle attitude error is reduced.

The rest of the paper is organized as follows: in Section 2, the principle and navigation algorithm of traditional single-axis rotation modulation are introduced. In Section 3, the RM scheme of the two-rotation mechanism is firstly proposed, then the rotation modulation theory of the two-rotation mechanism is discussed in detail, and the principle of the navigation solution of the new scheme is introduced. In Section 4, the effectiveness of the proposed monitoring method is verified through simulation and experiment. Finally, the conclusion is drawn in Section 5.

## 2. MEMS Single-Axis Rotating Inertial Navigation System

### 2.1. Principle of Single-Axis Rotation Modulation Error Suppression

**Figure 1:**
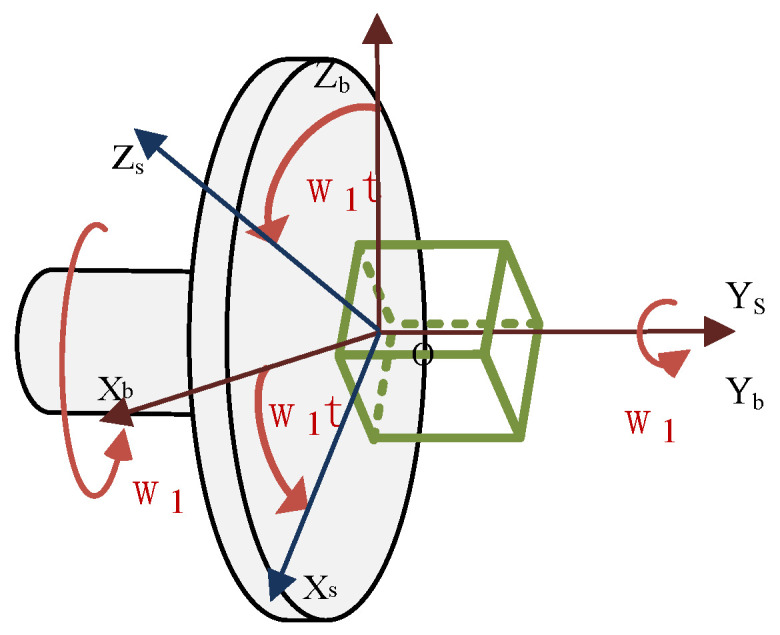
Microinertial measurement schematic diagram of uniaxial rotation modulation principle.

**Figure 2:**
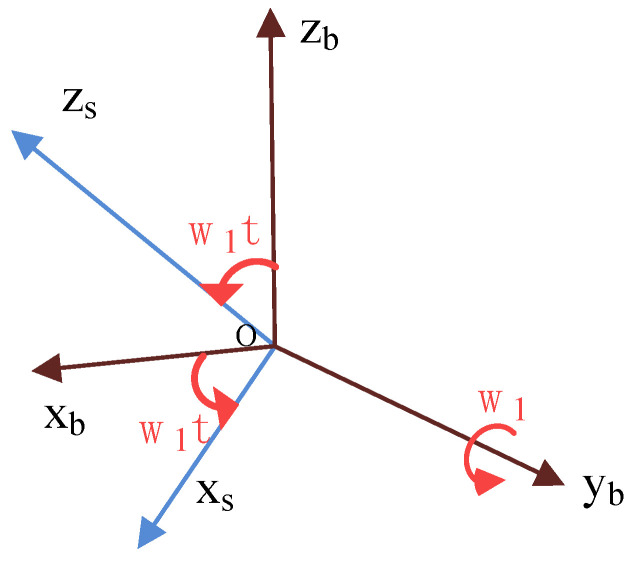
The b-frame and s-frame transformation diagram.

The constant error of MEMS inertial sensors is defined as a fixed and constant error after each start-up. It means that in the process of data processing, the quantitative compensation of the precalibration results cannot completely compensate for the constant error. You need to compensate for the real-time error each time the sensor is turned on; otherwise, the error will accumulate over time. Assuming that the IMU reference frame ($ox_{s}y_{s}z_{s}$) coincidentally with the carrier coordinate system ($ox_{b}y_{b}z_{b}$) at the initial moment, the IMU is controlled by a motor to continuously rotate around the Y-axis at a constant angular velocity ω, as shown in Figure 1. transformation diagram of b-frame and s-frame is shown in Figure 2.

$C_{b}^{s}$, the transformation matrix shown in Figure 2 between the carrier coordinate system and the IMU coordinate system, is an orthogonal matrix. Then, the transformation matrix shown in Figure 2 between the IMU coordinate system and the carrier coordinate system at time t can be expressed as follows:

(1) $$C_{s}^{b}={\left(C_{b}^{s}\right)}^{-1}={\left[C_{b}^{s}\right]}^{T}={\left[\begin{array}{ccc} cos\omega t & 0 & -sin\omega t\\ 0 & 1 & 0\\ sin\omega t & 0 & cos\omega t \end{array}\right]}^{T}=\left[\begin{array}{ccc} cos\omega t & 0 & sin\omega t\\ 0 & 1 & 0\\ -sin\omega t & 0 & cos\omega t \end{array}\right]$$

In a navigation system, it is assumed that the vehicle coordinate system and the navigation system coincide in order to simplify the problem. It means $C_{b}^{n}=I$. According to the relative angular positions of the IMU coordinate system and carrier coordinate system depicted in Figure 1, the modulation forms of the constant drift of the gyroscope and in the navigation coordinate system can be obtained, in the process of continuous clockwise rotation of the IMU at time t.

(2) $$\begin{array}{l} \delta \omega_{is}^{b}=\varepsilon^{b}=C_{s}^{b}\varepsilon^{s}\\ =\left[\begin{array}{ccc} cos\omega t & 0 & sin\omega t\\ 0 & 1 & 0\\ -sin\omega t & 0 & cos\omega t \end{array}\right]\left[\begin{array}{c} \varepsilon_{x}^{s}\\ \varepsilon_{y}^{s}\\ \varepsilon_{z}^{s} \end{array}\right]=\left[\begin{array}{c} \varepsilon_{x}^{s}cos\omega t+\varepsilon_{y}^{s}sin\omega t\\ \varepsilon_{y}^{s}\\ -\varepsilon_{x}^{s}sin\omega t-\varepsilon_{y}^{s}cos\omega t \end{array}\right] \end{array}$$

The constant error of the micro accelerometer is modulated as follows:

(3) $$\begin{array}{l} \delta f_{s}^{b}=\nabla^{b}=C_{s}^{b}\nabla^{s}\\ =\left[\begin{array}{ccc} cos\omega t & 0 & sin\omega t\\ 0 & 1 & 0\\ -sin\omega t & 0 & cos\omega t \end{array}\right]\left[\begin{array}{c} \nabla_{x}^{s}\\ \nabla_{y}^{s}\\ \nabla_{z}^{s} \end{array}\right]=\left[\begin{array}{c} \nabla_{x}^{s}cos\omega t+\nabla_{z}^{s}sin\omega t\\ \nabla_{y}^{s}\\ -\nabla_{x}^{s}sin\omega t+\nabla_{z}^{s}cos\omega t \end{array}\right] \end{array}$$

As can be seen from Equations (2) and (3), the deviation of the inertial devices in the X and Z directions is adjusted into periodic change signals due to the rotation of the IMU around the Y-axis. The periodic signals obtained after rotation in the X- and Z-axes are superimposed in one period, and the final cumulative error is zero, which ensures that the navigation accuracy is not greatly affected. At the same time, the deviation of the inertial device in the direction of the rotation axis is not modulated, and the errors of attitude, velocity, and position caused by such errors in the solution of the single-axis rotational inertial navigation system still accumulate over time. Therefore, the error modulation technique can only adjust the constant deviation of the inertial device in the direction perpendicular to the rotation axis.

From the above analysis, it can be inferred that the error modulation self-compensation technology rotates the IMU through a specific rotation scheme so that the error integral caused by the constant error term in the error propagation equation of the gyroscope and accelerometer is zero or close to zero, which reduces the accumulation of systematic errors and improves the navigation accuracy.

### 2.2. Principle of Single-Axis Rotation Modulation Navigation Algorithm

**Figure 3:**
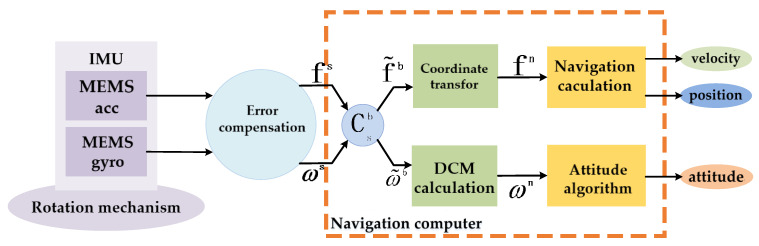
Schematic diagram of single-axis rotation modulation navigation solution process.

Therefore, the structure of inertial navigation systems using error modulation technology has changed. The existence of a rotating mechanism leads to the gyroscope and accelerometer no longer being fixated with the carrier, but the strapdown algorithm is still used to solve the problem. A schematic diagram of the single-axis rotation modulation navigation solution process is shown in Figure 3.

In Figure 3, MEMS inertial navigation system is mainly composed of IMU and navigation computer. Its working principle is as follows: firstly, the IMU composed of three micro gyroscopes and three micro accelerometers is installed on the rotating platform to measure the motion information in real time, and then the required navigation information is processed by the navigation computer after being compensated by existing error compensation model. IMU should be consistent with the axes of the carrier coordinate system and affixed to the vehicle, and at the same time, the MEMS gyroscopes and MEMS accelerometers should be mounted on three axes measuring the angular velocities and accelerations in the longitudinal, lateral, and vertical directions of the navigation coordinate system. However, values measured by MEMS gyroscopes and MEMS accelerometers comprise the data of the rotating coordinate system. After the transformation of the coordinate system, the sensor information under the rotation system is converted to information under the carrier coordinate system. Finally, the attitude, speed, and position under the navigation system are calculated by the navigation computer.

Through analysis, it can be derived that the above scheme improves the accuracy of traditional strapdown inertial navigation, while the constant error of the rotation axis is not modulated, and the constant error model of the traditional strapdown inertial navigation system is still accumulated over time in the navigation solution. This limits the accuracy improvement of the system. In the high-rotation and highly dynamic environment, it is necessary to design a new scheme to achieve the purpose of improving the reliability and accuracy of the system in the finite volume.

## 3. The Proposed Compound Rotation MEMS Inertial Navigation System

### 3.1. Compound Rotation Modulation System

#### 3.1.1. Principle of Compound Rotation Modulation System

**Figure 4:**
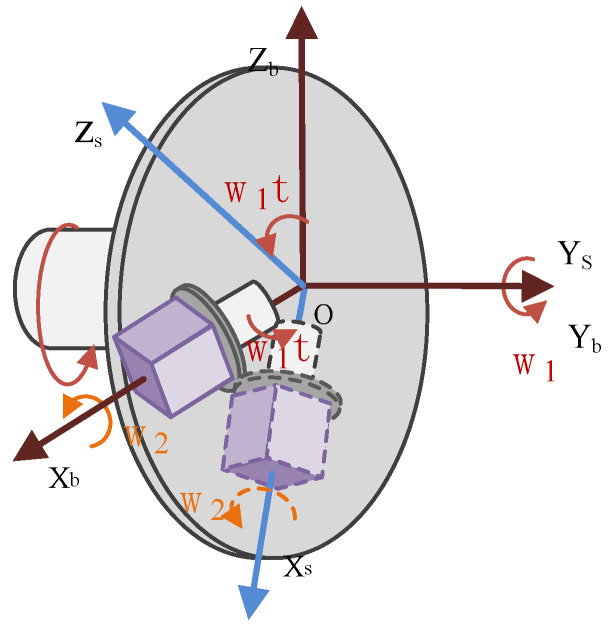
Schematic diagram of biaxial rotating IMU configuration.

Due to the complex structure, large volume, and limited storage space, the traditional dual-axis rotation modulation system is not suitable for conventional missile environments. In this case, the compound rotation modulation scheme was designed in which the IMU performed periodic compound symmetric rotation to offset the constant errors in the three axes by two motors operating independently at the same time. A schematic diagram of biaxial rotating IMU configuration is shown in Figure 4.

In Figure 4, one IMU is placed on rotating platforms, combined with a small rotating platform and strapdown with the large rotating platform. The IMU rotates around the axis of the smaller rotating platform while following the rotation of the larger rotating platform.

**Figure 5:**
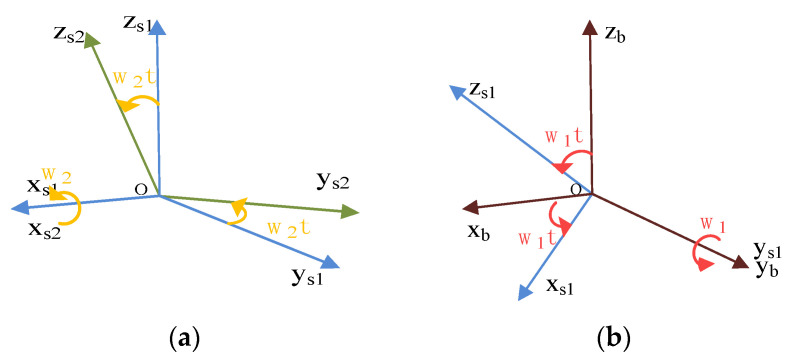
Compound rotation modulation system coordinate transformation diagram: (**a**) b-frame and s1-frame transformation diagram; (**b**) s1-frame and s2-frame transformation diagram.

The IMU is driven by a small rotating platform and rotates around the axis of rotation of a large rotating platform. IMU outputs a signal in an s2-frame, consisting of three gyroscopes and three accelerometers. The relative position relationship between s1-frame and s2-frame is shown in Figure 5. The relative angular position relationship between s1-frame and b-frame is shown in Figure 5 as well. While the large rotating mechanism rotates at an angular rate of $\omega_{1}$, the small rotating mechanism rotates at an angular rate of $\omega_{2}$.

The transformation relationship between s1-frame and b-frame, shown in Figure 5, can be described by a rotation transformation matrix $C_{s1}^{b}$, and the direction cosine matrix $C_{s1}^{b}$ could be expressed as follows:

(4) $$C_{s1}^{b}=\left[\begin{array}{ccc} cos\omega_{1}t & 0 & sin\omega_{1}t\\ 0 & 1 & 0\\ -sin\omega_{1}t & 0 & cos\omega_{1}t \end{array}\right]$$

The transformation relationship between s1-frame and b-frame, shown in Figure 5, can be described by a rotation transformation matrix $C_{s2}^{s1}$, and the direction cosine matrix $C_{s2}^{s1}$ could be expressed as follows:

(5) $$C_{s2}^{s1}=\left[\begin{array}{ccc} 1 & 0 & 0\\ 0 & cos\omega_{2}t & -sin\omega_{2}t\\ 0 & sin\omega_{2}t & cos\omega_{2}t \end{array}\right]$$

For the IMU, the following formula is obtained according to the matrix chain multiplication rule:

(6) $$\begin{array}{l} C_{s2}^{b}=C_{s1}^{b}C_{s2}^{s1}\\ =\left[\begin{array}{ccc} cos\omega_{1}t & 0 & sin\omega_{1}t\\ 0 & 1 & 0\\ -sin\omega_{1}t & 0 & cos\omega_{1}t \end{array}\right]\left[\begin{array}{ccc} 1 & 0 & 0\\ 0 & cos\omega_{2}t & -sin\omega_{2}t\\ 0 & sin\omega_{2}t & cos\omega_{2}t \end{array}\right] \end{array}$$

Based on the IMU, the constant error of the MEMS gyroscope is modulated as follows:

(7) $$\begin{array}{l} \delta w_{s2}^{b}=\varepsilon_{s2}^{b}=C_{s1}^{b}C_{s2}^{s1}\varepsilon^{s2}\\ =\left[\begin{array}{c} \varepsilon_{x}^{s}2cos\omega_{1}t+\frac{\varepsilon_{y}^{s2}}{2}cos\left(\omega_{1}+\omega_{2}\right)t-\frac{\varepsilon_{y}^{s2}}{2}cos\left(\omega_{1}-\omega_{2}\right)t+\frac{\varepsilon_{z}^{s2}}{2}sin\left(\omega_{1}+\omega_{2}\right)t+\frac{\varepsilon_{z}^{s2}}{2}sin\left(\omega_{1}-\omega_{2}\right)t\\ cos\omega_{2}t\varepsilon_{y}^{s2}-sin\omega_{2}t\varepsilon_{z}^{s2}\\ -\varepsilon_{x}^{s2}sin\omega_{1}t+\frac{\varepsilon_{y}^{s2}}{2}sin\left(\omega_{1}+\omega_{2}\right)t-\frac{\varepsilon_{y}^{s2}}{2}sin\left(\omega_{1}-\omega_{2}\right)t+\frac{\varepsilon_{z}^{s2}}{2}cos\left(\omega_{1}+\omega_{2}\right)t+\frac{\varepsilon_{z}^{s2}}{2}cos\left(\omega_{1}-\omega_{2}\right)t \end{array}\right]\\ =\left[\begin{array}{ccc} cos\omega_{1}t & \frac{cos\left(\omega_{1}+\omega_{2}\right)t-cos\left(\omega_{1}-\omega_{2}\right)t}{2} & \frac{sin\left(\omega_{1}+\omega_{2}\right)t+sin\left(\omega_{1}-\omega_{2}\right)t}{2}\\ 0 & cos\omega_{2}t & -sin\omega_{2}t\\ -sin\omega_{1}t & \frac{sin\left(\omega_{1}+\omega_{2}\right)t-sin\left(\omega_{1}-\omega_{2}\right)t}{2} & \frac{cos\left(\omega_{1}+\omega_{2}\right)t+cos\left(\omega_{1}-\omega_{2}\right)t}{2} \end{array}\right]\left[\begin{array}{c} \varepsilon_{x}^{s2}\\ \varepsilon_{y}^{s2}\\ \varepsilon_{z}^{s2} \end{array}\right] \end{array}$$

where $\varepsilon^{s2}={\left[\begin{array}{ccc} \varepsilon_{x}^{s2} & \varepsilon_{y}^{s2} & \varepsilon_{z}^{s2} \end{array}\right]}^{T}$ is the constant error of three gyroscopes of the IMU in the s2-frame. $\delta \omega_{s2}^{b}$ is the angular velocity error in the output information of the IMU in the b-frame. The constant error of the accelerometer of the IMU is modulated to the sum of sines and cosines at specific frequencies in the b-frame. The periodic component of each particular frequency can be canceled out after the integral operation of the whole period, and the error caused by the constant error accumulates to zero. According to the above analysis, the error caused by the gyroscope constant error in the b-frame accumulates to zero after the least common multiple of each period.

The constant error of MEMS accelerometers is modulated as follows:

(8) $$\begin{array}{l} \delta f_{s2}^{b}=\nabla_{s2}^{b}+w_{g}^{b}=C_{s1}^{b}C_{s2}^{s1}\nabla^{s2}+C_{s1}^{b}C_{s2}^{s1}w_{g}^{}\\ =\left[\begin{array}{c} \nabla_{x}^{s}2cos\omega_{1}t+\frac{\nabla_{y}^{s2}}{2}cos\left(\omega_{1}+\omega_{2}\right)t-\frac{\nabla_{y}^{s2}}{2}cos\left(\omega_{1}-\omega_{2}\right)t+\frac{\nabla_{z}^{s2}}{2}sin\left(\omega_{1}+\omega_{2}\right)t+\frac{\nabla_{z}^{s2}}{2}sin\left(\omega_{1}-\omega_{2}\right)t\\ cos\omega_{2}t\nabla_{y}^{s2}-sin\omega_{2}t\nabla_{z}^{s2}\\ -\nabla_{x}^{s2}sin\omega_{1}t+\frac{\nabla_{y}^{s2}}{2}sin\left(\omega_{1}+\omega_{2}\right)t-\frac{\nabla_{y}^{s2}}{2}sin\left(\omega_{1}-\omega_{2}\right)t+\frac{\nabla_{z}^{s2}}{2}cos\left(\omega_{1}+\omega_{2}\right)t+\frac{\nabla_{z}^{s2}}{2}cos\left(\omega_{1}-\omega_{2}\right)t \end{array}\right]\\ =\left[\begin{array}{ccc} cos\omega_{1}t & \frac{cos\left(\omega_{1}+\omega_{2}\right)t-cos\left(\omega_{1}-\omega_{2}\right)t}{2} & \frac{sin\left(\omega_{1}+\omega_{2}\right)t+sin\left(\omega_{1}-\omega_{2}\right)t}{2}\\ 0 & cos\omega_{2}t & -sin\omega_{2}t\\ -sin\omega_{1}t & \frac{sin\left(\omega_{1}+\omega_{2}\right)t-sin\left(\omega_{1}-\omega_{2}\right)t}{2} & \frac{cos\left(\omega_{1}+\omega_{2}\right)t+cos\left(\omega_{1}-\omega_{2}\right)t}{2} \end{array}\right]\left[\begin{array}{c} \nabla_{x}^{s2}\\ \nabla_{y}^{s2}\\ \nabla_{z}^{s2} \end{array}\right] \end{array}$$

where $\nabla_{2}^{s2}={\left[\begin{array}{ccc} \nabla_{x}^{s2} & \nabla_{y}^{s2} & \nabla_{z}^{s2} \end{array}\right]}^{T}$ is the constant error of three accelerometers of the IMU in the s2-frame. $\delta f_{s2}^{b}$ is the angular velocity error in the output information of the IMU in the b-frame. The principle of constant error modulation of the accelerometer of the IMU is the same as that of the gyroscope. The analysis shows that the error caused by the constant error of the accelerometer in the b-frame accumulates to zero after the least common multiple of each period.

#### 3.1.2. Scheme of Compound Rotation Modulation System

Rotation modulation offsets the constant error in one period by following a specially arranged rotation scheme. For novel configuration of the IMUs, the corresponding rotation scheme design is required. According to the analysis in the previous chapter, we can obtain the constant error modulation mechanism of the IMU as follows:

(9) $$\delta \omega_{s2}^{b}=\left[\begin{array}{ccc} cos\omega_{1}t & \frac{cosat-cosbt}{2} & \frac{sinat+sinbt}{2}\\ 0 & cos\omega_{2}t & -sin\omega_{2}t\\ -sin\omega_{1}t & \frac{sinat-sinbt}{2} & \frac{cosat+cosbt}{2} \end{array}\right]\left[\begin{array}{c} \varepsilon_{x}^{s2}\\ \varepsilon_{y}^{s2}\\ \varepsilon_{z}^{s2} \end{array}\right]$$

(10) $$\delta f_{s2}^{b}=\left[\begin{array}{ccc} cos\omega_{1}t & \frac{cosat-cosbt}{2} & \frac{sinat+sinbt}{2}\\ 0 & cos\omega_{2}t & -sin\omega_{2}t\\ -sin\omega_{1}t & \frac{sinat-sinbt}{2} & \frac{cosat+cosbt}{2} \end{array}\right]\left[\begin{array}{c} \nabla_{x}^{s2}\\ \nabla_{y}^{s2}\\ \nabla_{z}^{s2} \end{array}\right]$$

where $a=\omega_{1}+\omega_{2}$, $b=\omega_{1}-\omega_{2}$.

Assuming $\omega_{1}=\omega_{2}$ and substituting into the formula above, the formula becomes as follows:

(11) $$\begin{array}{l} \delta \omega_{s2}^{b}=\left[\begin{array}{ccc} cos\omega_{1}t & \frac{cos2\omega_{1}t-1}{2} & \frac{sin2\omega_{1}t+1}{2}\\ 0 & cos\omega_{1}t & -sin\omega_{1}t\\ -sin\omega_{1}t & \frac{sin2\omega_{1}t-1}{2} & \frac{cos2\omega_{1}t+1}{2} \end{array}\right]\left[\begin{array}{c} \varepsilon_{x}^{s2}\\ \varepsilon_{y}^{s2}\\ \varepsilon_{z}^{s2} \end{array}\right]\\ =\left[\begin{array}{c} cos\omega_{1}t\varepsilon_{x}^{s2}+\frac{cos2\omega_{1}t}{2}\varepsilon_{y}^{s2}+\frac{sin2\omega_{1}t}{2}\varepsilon_{z}^{s2}-\frac{1}{2}\varepsilon_{y}^{s2}+\frac{1}{2}\varepsilon_{z}^{s2}\\ cos\omega_{1}t\varepsilon_{y}^{s2}-sin\omega_{1}t\varepsilon_{z}^{s2}\\ -sin\omega_{1}t\varepsilon_{x}^{s2}+\frac{sin2\omega_{1}t}{2}\varepsilon_{y}^{s2}+\frac{cos2\omega_{1}t}{2}\varepsilon_{z}^{s2}-\frac{1}{2}\varepsilon_{y}^{s2}+\frac{1}{2}\varepsilon_{z}^{s2} \end{array}\right] \end{array}$$

From the above equation, we can infer that under the condition of $\omega_{1}=\omega_{2}$, there is still a component of constant error in the result of modulation error, and the constant error cannot be completely modulated.

(12) $$T=\left(2\pi /\omega_{1},2\pi /a,2\pi /\omega_{2},2\pi /b\right)$$

According to the basic principle of rotation modulation, T, which is the greatest common divisor of each sinusoidal period, is the period during which the constant error of IMU2 is modulated to zero completely.

(13) $$\int_{0}^{T}\delta \omega_{s2}^{b}dt=\int_{0}^{T}\left[\begin{array}{ccc} cos\omega_{1}t & \frac{cosat-cosbt}{2} & \frac{sinat+sinbt}{2}\\ 0 & cos\omega_{2}t & -sin\omega_{2}t\\ -sin\omega_{1}t & \frac{sinat-sinbt}{2} & \frac{cosat+cosbt}{2} \end{array}\right]\left[\begin{array}{c} \varepsilon_{x}^{s2}\\ \varepsilon_{y}^{s2}\\ \varepsilon_{z}^{s2} \end{array}\right]dt=\left[\begin{array}{c} 0\\ 0\\ 0 \end{array}\right]$$

(14) $$\int_{0}^{T}\delta f_{s2}^{b}dt=\int_{0}^{T}\left[\begin{array}{ccc} cos\omega_{1}t & \frac{cosat-cosbt}{2} & \frac{sinat+sinbt}{2}\\ 0 & cos\omega_{2}t & -sin\omega_{2}t\\ -sin\omega_{1}t & \frac{sinat-sinbt}{2} & \frac{cosat+cosbt}{2} \end{array}\right]\left[\begin{array}{c} \nabla_{x}^{s2}\\ \nabla_{y}^{s2}\\ \nabla_{z}^{s2} \end{array}\right]dt=\left[\begin{array}{c} 0\\ 0\\ 0 \end{array}\right]$$

**Figure 6:**
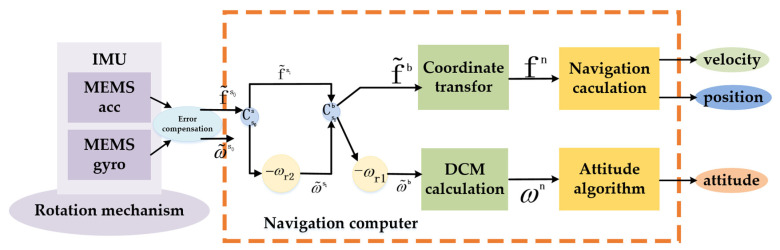
Compound rotation modulation navigation algorithm.

Compared with the traditional strapdown algorithm, the main navigation algorithm still adopts the traditional strapdown navigation algorithm in the composite rotation modulation scheme, but the influence of rotation modulation on the angular rate introduced in the navigation solution should be considered in the navigation solution. The compound rotation modulation navigation algorithm is shown in Figure 6.

Using the composite rotation modulation scheme, the inertial sensor parameters measured by IMU in the s0-frame are the motion state parameters of IMU rather than the inertial measurement parameters of the carrier that are required in the final navigation calculation. Therefore, the modulated angular velocity provided by the rotating mechanism should be eliminated during the navigation calculation. In Figure 6, $\omega_{r1}$ refers to the modified modulation angular rate during the transformation of the first-level coordinate system, and $\omega_{r2}$ refers to the modified modulation angular rate during the transformation of the second-level coordinate system.

(15) $${\tilde{\omega }}^{s_{0}}=C_{s_{1}}^{s_{0}}{\tilde{\omega }}^{s_{1}}-\omega_{r2}$$

(16) $${\tilde{\omega }}^{b}=C_{s_{0}}^{b}{\tilde{\omega }}^{s_{0}}-\omega_{r1}$$

where $\omega_{r2}={\left[\begin{array}{ccc} \omega_{2} & 0 & 0 \end{array}\right]}^{T}$ and $\omega_{r1}={\left[\begin{array}{ccc} 0 & \omega_{1} & 0 \end{array}\right]}^{T}$. $\omega_{2}$ is the modulation angular rates of the second rotating mechanism, and $\omega_{1}$ is the modulation angular rates of the first rotating system. After the coordinate system transformation and the deduction of the modulation angular rate, the angular rate of the b-frame is finally calculated. It is worth noting that at this time, the constant error of inertia information in the b-frame is modulated as a periodic signal and accumulates to 0 in one period. Based on this principle, the constant error is canceled out, and the divergence of error is suppressed in the following navigation solution.

### 3.2. Feasibility Analysis

In this section, by comparing the performance of the proposed composite rotation modulation scheme with the traditional single-axis rotation modulation scheme, the error suppression performance and correctness of the theory of the proposed composite rotation modulation scheme are evaluated by simulation. The bias of the three accelerometers in the IMU was set to 2 mg, and the bias of the three accelerometers in the IMU was set to 24°/h, as shown in Table 1.

According to theoretical requirements, the rotating speed of the first rotating mechanism was selected as 120°/s, and that of the second rotating mechanism was selected as 60°/s in the compound rotation modulation simulation experiment. The modulation angular rate was selected as 120°/s for the single-axis rotation modulation scheme. Table 2 summarizes the theoretical values of the parameters under the two rotational modulation schemes.

**Figure 7:**
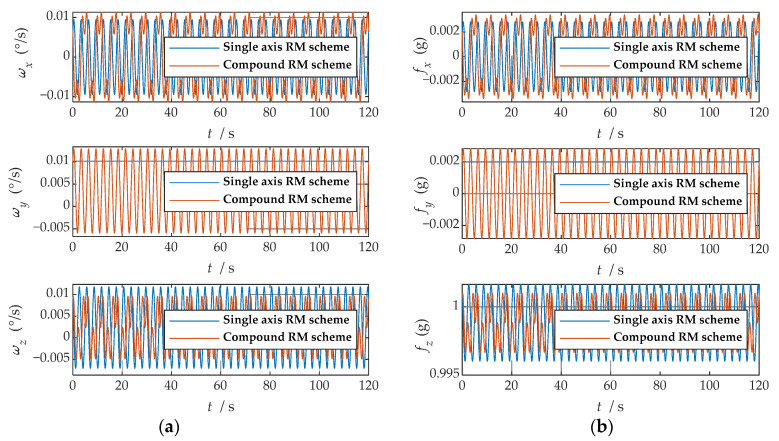
The navigation information of the b-frame obtained after coordinate system transformation: (**a**) angular velocity information; (**b**) specific force information.

**Figure 8:**
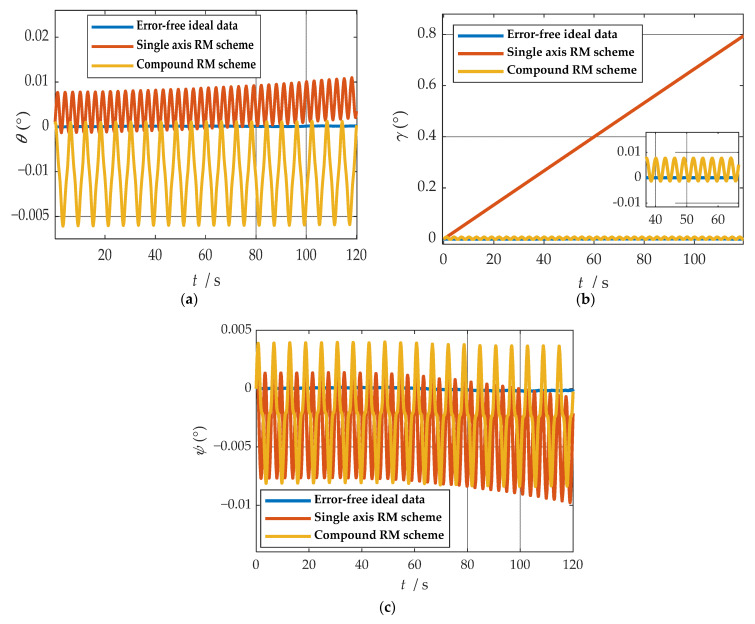
Comparison of attitude solution results: (**a**) comparison of calculation results of yaw angle; (**b**) comparison of calculation results of roll angle; (**c**) comparison of calculation results of pitch angle.

**Figure 9:**
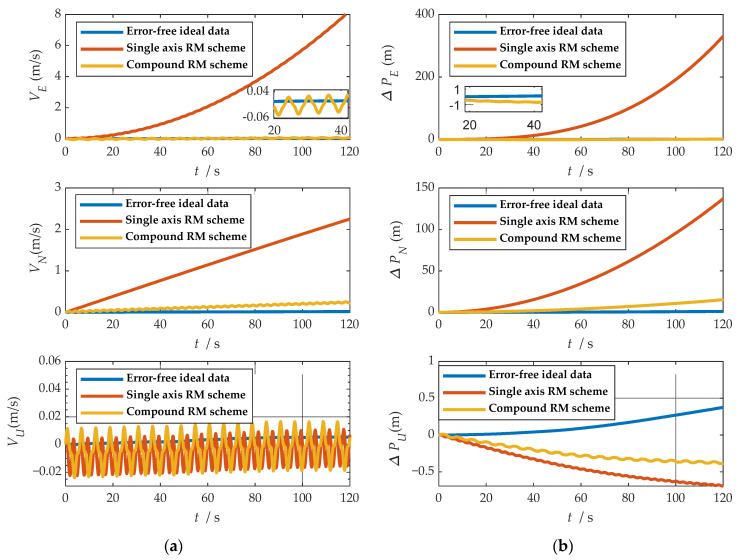
Comparison of velocity and position results in three directions: (**a**) comparison of velocity results in three directions; (**b**) comparison of position results in three directions.

First, the error was set according to Table 1, and the IMU data generator generated the IMU output data under the two rotation modulation schemes, respectively. Second, the two sets of data were, respectively, transformed to the b-frame by coordinate system transformation. The angular rate and specific force information of the b-frame obtained after coordinate transformation is shown in Figure 7.

where $\omega_{x}$, $\omega_{y}$ and $\omega_{z}$ represent the angular velocities of the X-, Y-, and Z-axes. $f_{x}$, $f_{y}$ and $f_{z}$ represent the specific forces of X-, Y-, and Z-axes, respectively. Figure 7 shows the angular rates of the two schemes and the output results of the accelerometer under the b-frame. The blue line represents the data under the single-axis rotation modulation scheme, and the red line represents the data under the compound rotation modulation scheme. It can be observed from Figure 7 that the constant errors of the X- and Z-axes of the first IMU are modulated into periodic signals that can be canceled within a cycle, but the constant errors of the Y-axis are not modulated. The constant error of the three axes can be modulated into a periodic signal under the compound rotation modulation scheme. However, the modulation cycle of the new rotation modulation scheme is longer than that of the single-axis rotation modulation scheme, and the modulation cycle of the new scheme is twice as long as that of the single-axis rotation modulation scheme when the current modulation angular velocity is selected. The attitude angle information obtained through navigation is compared, as shown in Figure 8. The velocity and position results in three directions are compared, as shown in Figure 9. The maximum errors of navigation parameters are summarized in Table 3.

where θ represents yaw angle. γ represents yaw angle. ψ represents yaw angle. $\phi_{E}$, $\phi_{N}$ and $\phi_{U}$ represent eastward, northward, and upward misalignment angle, respectively.

where $V_{E}$, $V_{N}$ and $V_{U}$ represent eastward, northward and upward speeds, respectively. $P_{E}$, $P_{N}$ and $P_{U}$ represent eastward, northward and upward speeds, respectively. $\delta V_{E}$, $\delta V_{N}$ and $\delta V_{U}$ represent eastward, northward and upward speeds, respectively. $\delta P_{E}$, $\delta P_{N}$ and $\delta P_{U}$ represent eastward, northward and upward speeds, respectively.

**Table 3:** Maximum errors between two rotational modulation schemes comparison.

Maximum Error	Single Axis RM Scheme	Compound RM Scheme
$\phi_{E}$ (″)	11.7772	0.5693
$\phi_{N}$ (″)	2869.3556	0.0784
$\phi_{U}$ (′)	0.1156	0.0047
$\delta V_{E}$ (m/s)	8.2028	0.0669
$\delta V_{N}$ (m/s)	2.2324	0.2163
$\delta V_{U}$ (m/s)	0.0020	0.0002
$\delta P_{E}$ (m)	135.8699	14.1145
$\delta P_{N}$ (m)	398.1353	1.0460
$\delta P_{U}$ (m)	1.0610	0.7617

As shown in Figure 8, when the single-axis rotation modulation scheme is applied, the pitch angle and yaw angle do not diverge significantly. However, the constant error of the roll axis is not modulated, and the roll angle error calculated by this scheme accumulates over time. When the compound rotation modulation scheme is applied, the roll axis error is effectively modulated, and the offset degree of pitch angle and yaw angle is smaller than that of the traditional scheme. As is shown in Figure 9, in the case of the new rotation modulation scheme, the error accumulation of each velocity and position calculated by the navigation solution is inhibited to some extent. However, at the same time, we can infer from Figure 8 and Figure 9 that the modulation cycle of the information obtained by the navigation solution of the uniaxial rotation modulation scheme is shorter, but the error drift is significant, while the error modulation amplitude of the information obtained by the navigation solution of the new scheme is larger. The modulation period is longer, but the accuracy is improved, and the error divergence of the new modulation scheme is effectively suppressed in the static state. The simulation results verify the feasibility of the composite rotation modulation scheme effectively, as shown in Table 3.

## 4. Simulation and Experimental Results and Analysis

### 4.1. Simulation Results of Rotary Modulation Scheme

#### 4.1.1. Attitude Motion Simulation

In this section, IMU data when the system is moving are generated through simulation to compare the error suppression performance of the proposed composite rotation modulation scheme and the traditional single-axis rotation modulation scheme under the yaw angle motion environment. The error parameters of IMU are shown in Table 4. The motion state is set in Table 5.

**Figure 10:**
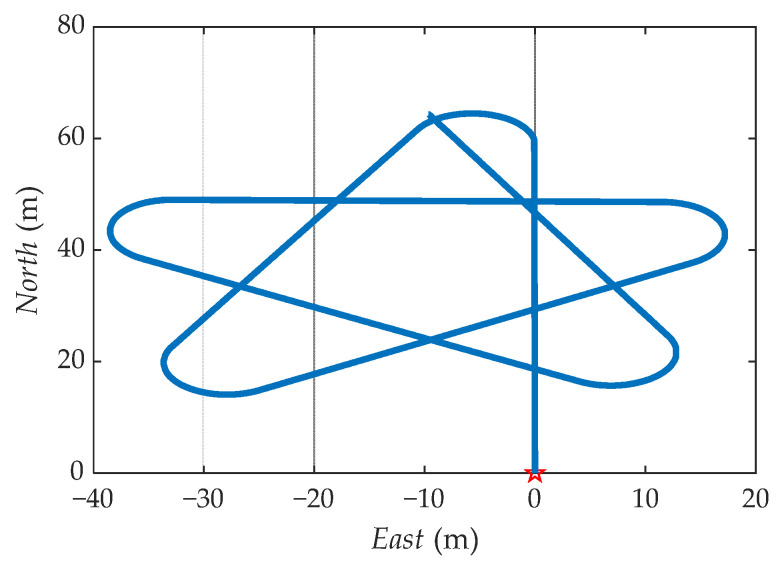
The simulation sets the original trajectory.

**Figure 11:**
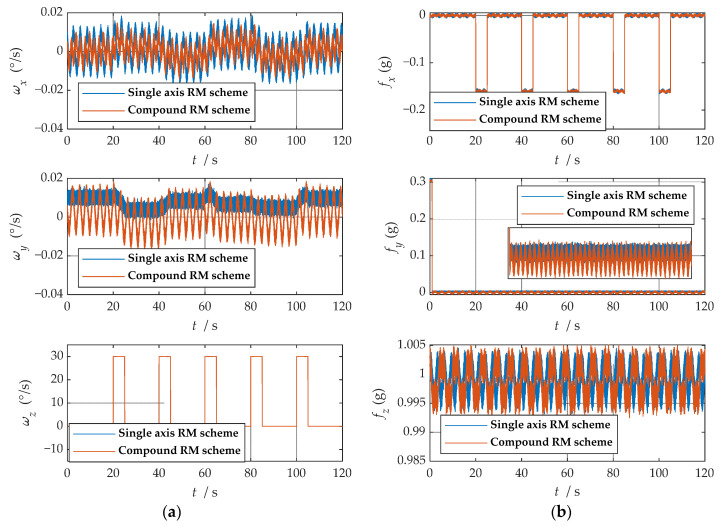
The navigation information of the b-frame obtained after coordinate system transformation: (**a**) angular velocity information; (**b**) specific force information.

The ideal trajectory without error generated by the simulation is shown in Figure 10. The navigation information of b-frame obtained after coordinate transformation is shown in Figure 11.

**Figure 12:**
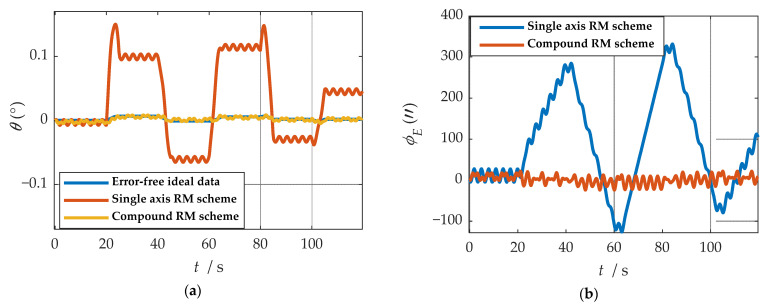
The navigation results comparison of the rotation scheme: (**a**) comparison of calculation results of yaw angle; (**b**) comparison of calculation results of east misalignment angle.

**Figure 13:**
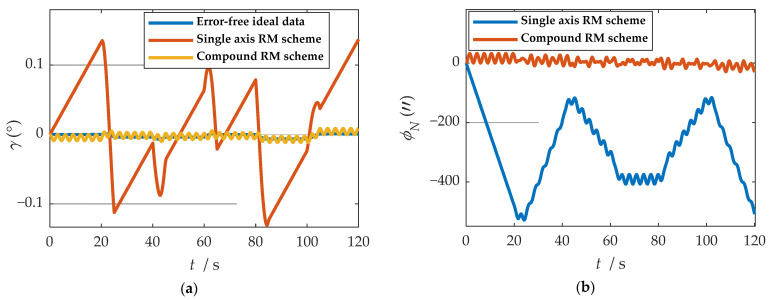
The navigation results comparison of the rotation scheme: (**a**) comparison of calculation results of roll angle; (**b**) comparison of calculation results of north misalignment angle.

**Figure 14:**
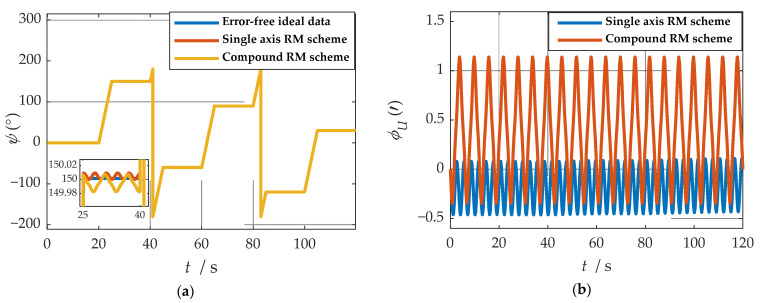
The navigation results comparison of the rotation scheme: (**a**) comparison of calculation results of pitch angle; (**b**) comparison of calculation results of up misalignment angle.

**Figure 15:**
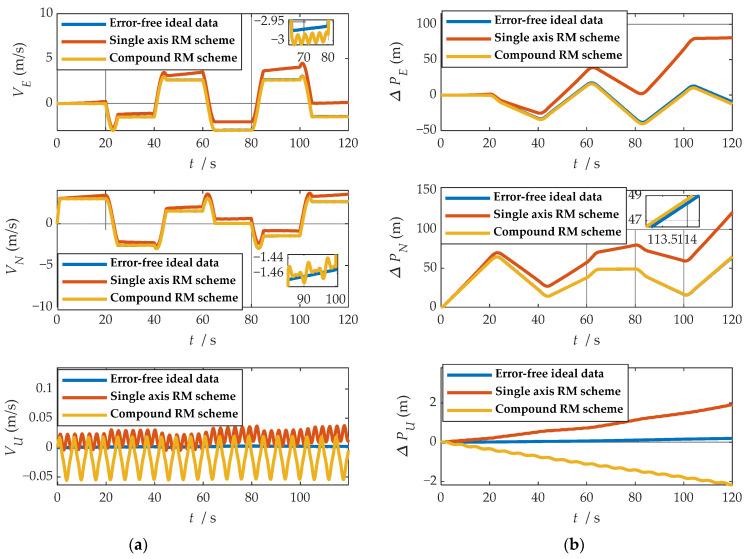
Comparison of velocity and position results in three directions: (**a**) comparison of velocity results in three directions; (**b**) comparison of position results in three directions.

**Figure 16:**
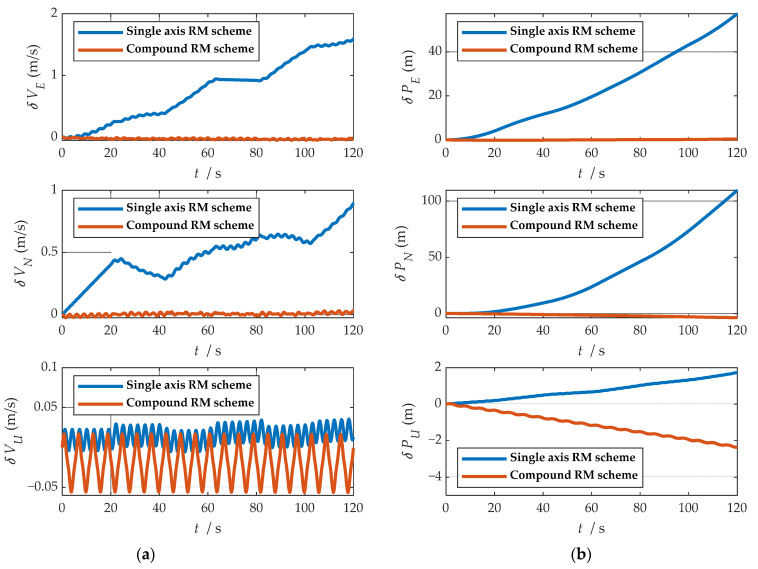
Comparison of velocity and position error in three directions: (**a**) comparison of velocity error in three directions; (**b**) comparison of position error in three directions.

**Figure 17:**
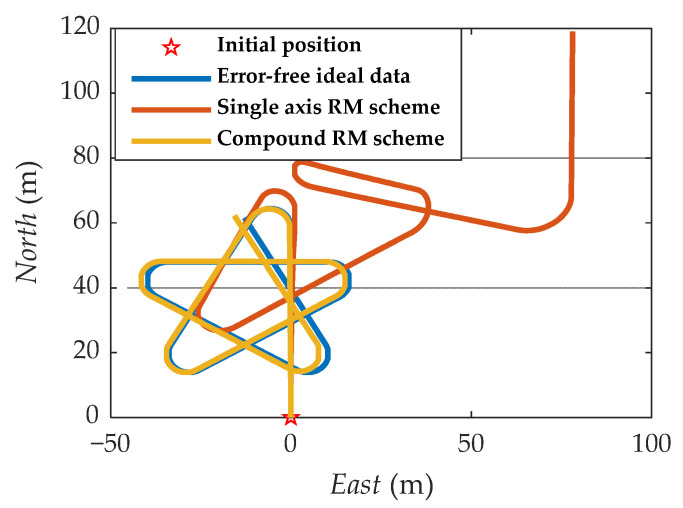
Comparison of trajectories obtained by navigation solution.

It is easy to detect that under the compound rotation modulation scheme, the IMU’s rotation axis is also modulated under the b-frame. As can be seen from Figure 11, the modulation result is periodic variation based on the ideal value. Consistent with the theoretical analysis, the constant error is modulated into an error that accumulates to zero over a period. The navigation results comparison of the rotation scheme is shown in Figure 12, Figure 13, Figure 14, Figure 15 and Figure 16. Comparison of trajectories obtained by navigation solution is shown in Figure 17. The maximum errors of navigation parameters are summarized in Table 6.

According to the results of navigation calculation, when the pitch angle and roll angle change, the traditional uniaxial rotation modulation changes at the moments of 20 s, 40 s, 60 s, 80 s, and 100 s in Figure 12, Figure 13, Figure 14, Figure 15 and Figure 16. The constant error of the roll axis accumulates over time because the roll axis is completely unmodulated. Especially when the attitude changes, it can be observed from Figure 12 and Figure 13 that the new rotation modulation scheme shows a good attitude error modulation effect. It can be observed from Figure 15 and Figure 16 that the compound rotation modulation scheme has obvious speed and position error suppression effects in the eastward and northward directions, compared with traditional rotation modulation. It can be observed from Figure 17 that the new rotary modulation scheme effectively improves the trajectory tracking capability. The maximum error in the altitude direction increases because the modulation scheme accumulates errors in the $Z_{b}$ direction. We can derive from Table 6 that maximum errors of the navigation parameter are reduced by an order of magnitude or two.

#### 4.1.2. High Spin Motion Simulation

In this section, data are generated through simulation to compare the error suppression performance of the proposed composite rotation modulation scheme and the traditional single-axis rotation modulation scheme under a high spin motion environment. The error parameters of IMU are shown in Table 4. The motion state is set to roll at a high speed of 30 rad/s, and the total simulation time is 120 s.

**Figure 18:**
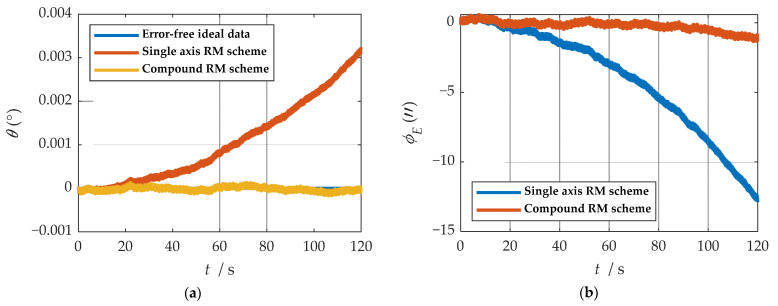
The navigation results comparison of the rotation scheme: (**a**) comparison of calculation results of yaw angle; (**b**) comparison of calculation results of east misalignment angle.

**Figure 19:**
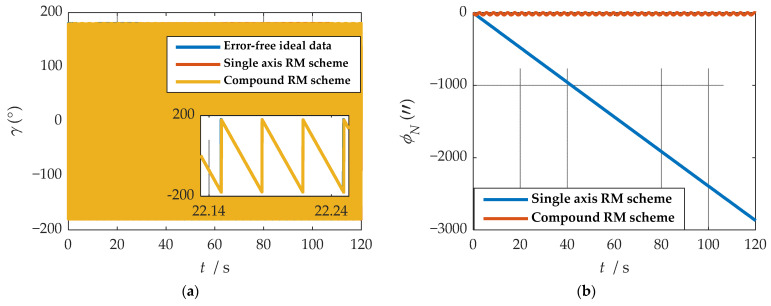
The navigation results comparison of the rotation scheme: (**a**) comparison of calculation results of roll angle; (**b**) comparison of calculation results of north misalignment angle.

**Figure 20:**
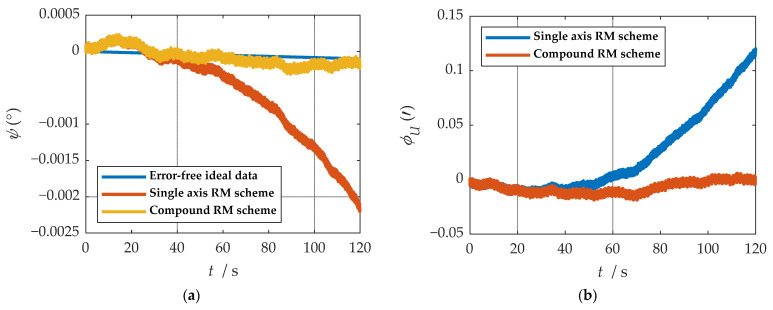
The navigation results comparison of the rotation scheme: (**a**) comparison of calculation results of pitch angle; (**b**) comparison of calculation results of up misalignment angle.

**Figure 21:**
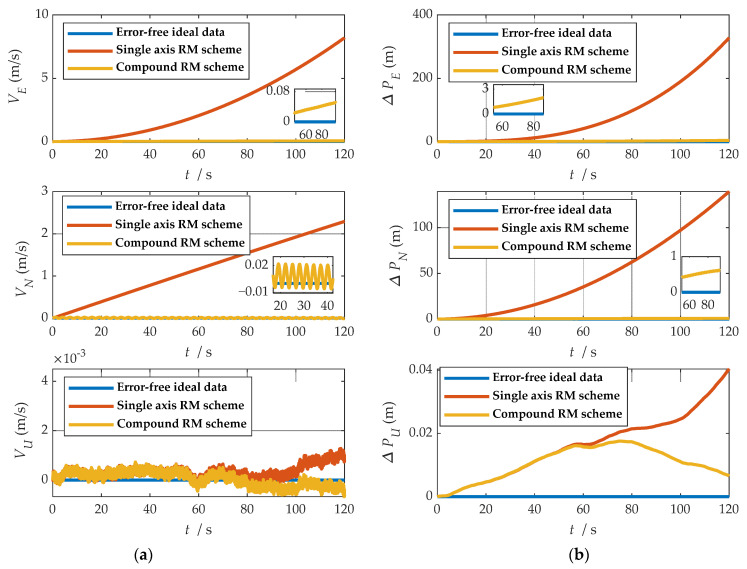
Comparison of velocity and position results in three directions: (**a**) comparison of velocity results in three directions; (**b**) comparison of position results in three directions.

Comparisons of calculation results of attitude are shown in Figure 18a, Figure 19a and Figure 20a. Comparisons of calculation results of misalignment angle are shown in Figure 18b, Figure 19b and Figure 20b. Comparison of velocity and position results in eastward, northward, and upward are shown in Figure 21.

In the high rotation state, using the traditional rotation modulation scheme, the two directions perpendicular to the modulation axis of the constant error are effectively suppressed, the modulation axis of the constant error is not suppressed, and error divergence is the most significant. The pitch and yaw angles also contain errors since the error is decomposed in the three axes of the n-frame after the coordinate transformation.

As shown in Figure 21, under the traditional rotation modulation scheme, the constant error in the direction of the modulation axis is not suppressed. In the case of high rotation, the error divergence of eastward and skyward is lower than that of the general case. Additionally, the velocity and position error divergences of the north direction are more significant. The maximum error values of each navigation parameter are summarized in Table 7.

Figure 18, Figure 19 and Figure 20 show that the compound rotation modulation scheme inhibits the continuing divergence of the misalignment angle caused by the constant error. As shown in Table 7, except for the height error that is reduced by half, the error of other parameters caused by the constant value error is reduced by one to four orders of magnitude. In particular, the divergence of the roll angle error is completely suppressed, and the accuracy has been improved by four orders of magnitude.

### 4.2. Experimental Results of Rotary Modulation Scheme

**Figure 22:**
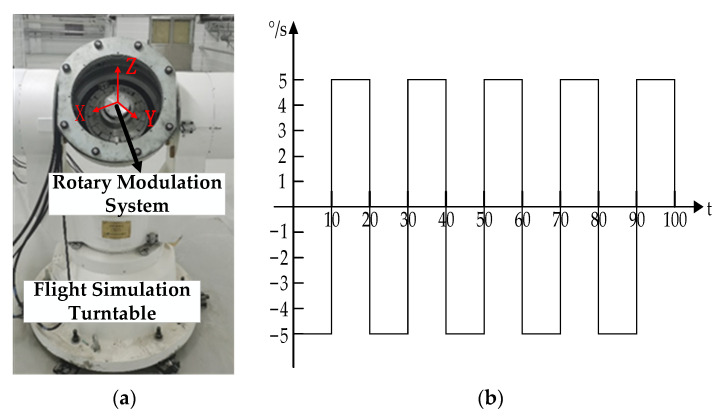
The experimental equipment: (**a**) the experimental equipment; (**b**) configuration of experimental swing state.

In order to verify the effectiveness of the implementation of the new rotation scheme proposed in this study, two experiments were carried out on a flight simulation turntable. The experimental equipment is shown in Figure 22a, which is composed of the new rotary modulation navigation system with a sampling rate of 500 Hz and a laptop computer to read experimental data. Technical parameters of the flight simulation turntable are shown in Table 8. Parameters of the IMU in the experiment are shown in Table 9.

In order to verify the inhibition effect of the compound rotation scheme on the divergence of roll angle error caused by constant error, two groups of experiments were designed.

Experiment 1: The flight simulation turntable remained static, and the internal rotation modulation system rotated according to two rotation schemes, respectively. The rotation modulation effect was verified under the static state of the carrier, and the total duration of the experiment was 110 s.

Experiment 2: The flight simulation turntable was set with swing configuration as shown in Figure 22b, and the internal rotation modulation system rotated, respectively, according to two rotation schemes. In order to compare the error suppression effect of the two rotational modulation schemes under the rolling angle motion, the two rotational modulation schemes were applied to carry out a comparative experiment under the pre-designed carrier swing state, and the total duration of the experiment was 100 s.

#### 4.2.1. Experiment 1: Static Experiment on Rotary Table

**Figure 23:**
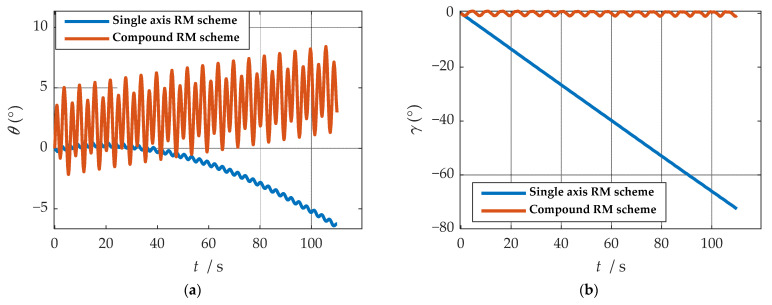

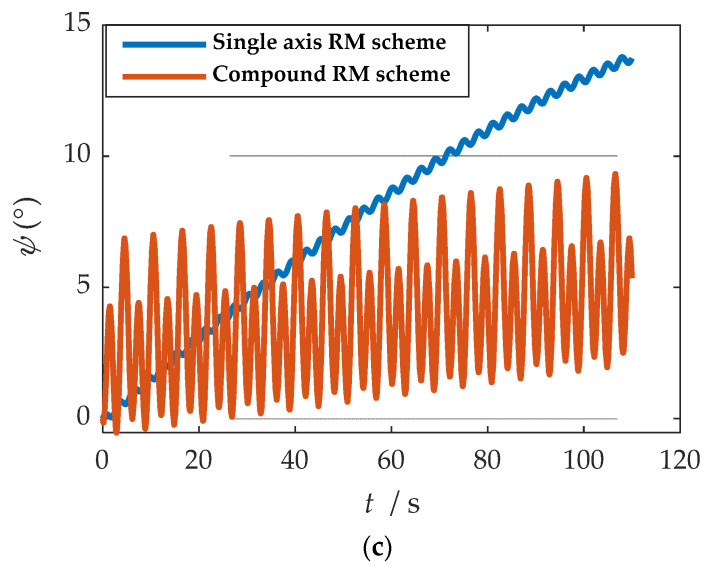
Comparison of attitude solution results: (**a**) comparison of calculation results of yaw angle; (**b**) comparison of calculation results of roll angle; (**c**) comparison of calculation results of pitch angle.

**Figure 24:**
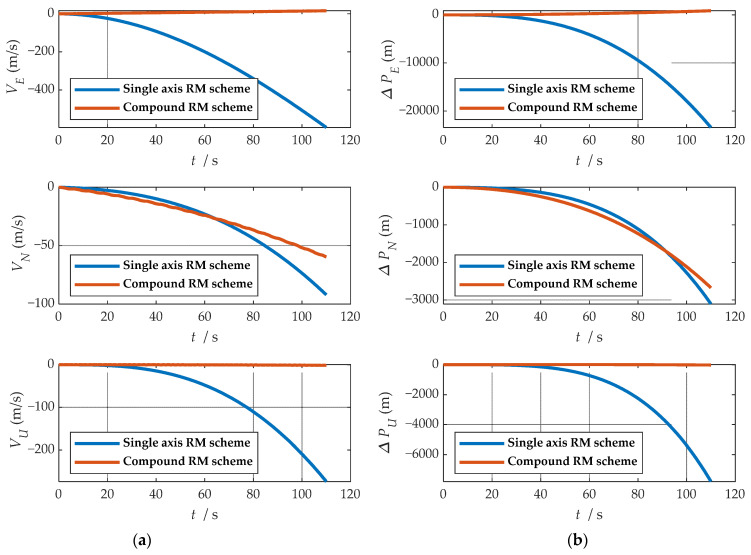
Comparison of velocity and position results in three directions: (**a**) comparison of velocity results in three directions; (**b**) comparison of position results in three directions.

After reading the data measured in the experiment, the navigation parameters were obtained through a navigation solution. The attitude solution results of the two schemes are shown in Figure 23. After navigation, the velocity and attitude parameters were calculated and are shown in Figure 24.

As shown in Table 10, in the experiment of 110 s, the error of all parameters decreases. In particular, the divergence of roll angle error is completely suppressed, and the accuracy is increased by two orders of magnitude. The eastward velocity and position error are greatly inhibited. The system’s roll axis points to the east; thus, the error divergence of velocity and position in the eastward in the traditional rotation scheme is larger than that in the other two directions in the experiment. In contrast, the eastward error is effectively suppressed under the compound rotation modulation scheme. The experimental results show that the new scheme presented in this paper can effectively suppress the error divergence of navigation parameters caused by the constant error in the direction of the modulation axis.

#### 4.2.2. Experiment 2: The Turntable Swayed

**Figure 25:**
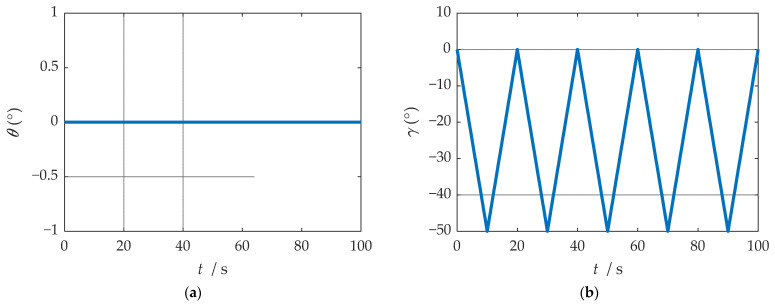

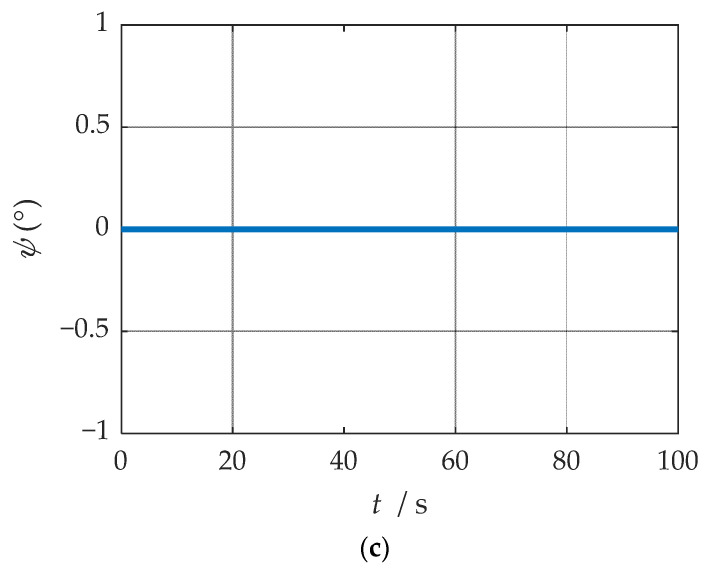
Ideal attitude solution results: (**a**) ideal results of yaw angle; (**b**) ideal results of roll angle; (**c**) ideal results of pitch angle.

**Figure 26:**
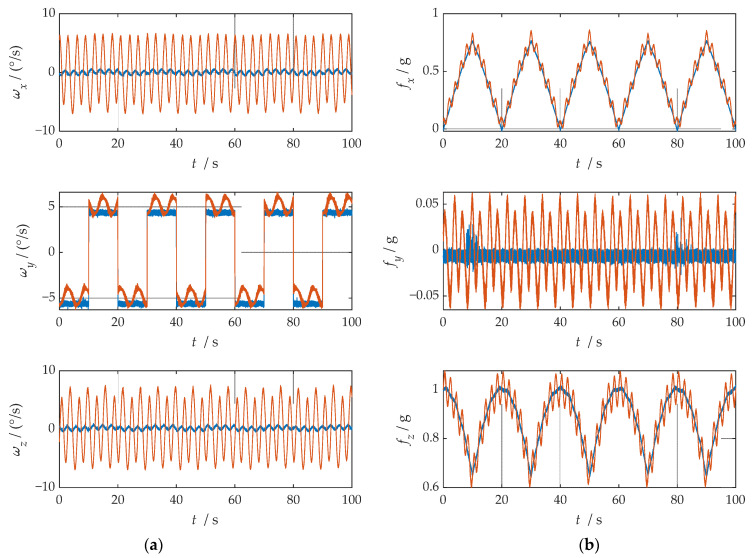
The navigation information of the b-frame obtained after coordinate system transformation: (**a**) angular velocity information; (**b**) specific force information.

According to the configuration set in the experiment, the ideal attitude results of the experiment are shown in Figure 25. In order to facilitate the experimental analysis, the configuration of the turntable was set as the regular angle change, but the regular angle change is easy to affect the precision judgment of the speed and position of the rotation modulation scheme; thus, the speed and position were not analyzed in this experiment. The navigation information of the b-frame obtained after coordinate transformation is shown in Figure 26.

**Figure 27:**
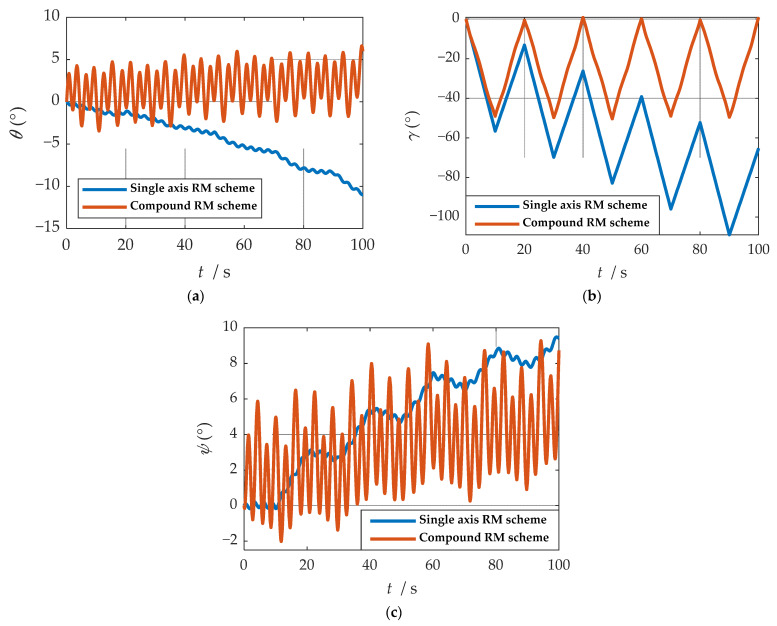
Comparison of attitude solution results: (**a**) comparison of calculation results of yaw angle; (**b**) comparison of calculation results of roll angle; (**c**) comparison of calculation results of pitch angle.

As can be observed from Figure 26, after coordinate system transformation in navigation solution, the IMU data of compound rotation modulation was calculated to a periodic signal with an estimated true value under b-frame. Additionally, the attitude solution results are shown in Figure 27. Maximum errors between two rotational modulation schemes comparison are shown in Table 11.

As shown in Figure 27, in the traditional rotation modulation scheme, because the constant error compensation is not compensated, the roll angle error is linearly divergent. Compared with the traditional rotational modulation scheme, the composite rotational modulation scheme can suppress the linear divergence caused by the constant error. The tracking ability of the compound rotation scheme is verified when the angle and angular velocity change. As shown in Table 11, in the experiment of 100 s, the angle error decreases. In particular, the divergence of roll angle error is completely suppressed, and the accuracy is increased by two orders of magnitude.

## 5. Conclusions

In this paper, by analyzing the single-axis rotation modulation error modulation model and the error propagation mechanism of navigation solution, it was found that single-axis rotation modulation cannot suppress the gyroscope constant value error in the direction of the rotation axis, which caused inaccurate attitude matrix calculation. In order to improve the navigation accuracy of microinertial navigation system and reduce the error of roll angle, a new rotation modulation scheme is proposed. This rotation modulation scheme changes the constant error propagation model by controlling the compound rotation of the IMU. Simulation and experimental results show that, compared with the traditional implementation, the proposed rotation modulation scheme can realize the constant error modulation of the roll direction, and the precision of the roll angle can be improved by two orders of magnitude. The experiment and simulation show that the new scheme can effectively suppress the divergence of the modulation axis error, and the navigation accuracy is improved under the new rotation modulation scheme.

## Figures and Tables

**Table 1:** Parameters of the sensors in simulation experiment.

Characteristics	Bias	Random Walk
Gyroscope (X-, Y-, Z-axis)	24°/h	0.001°/$\sqrt{h}$
Accelerometer (X-, Y-, Z-axis)	2 mg	1000 ug/$\sqrt{Hz}$

**Table 2:** Ideal parameters of two rotational modulation schemes.

Parameter after a Complete Rotation Cycle	Compound RM Scheme	Single Axis RM Scheme
Gyroscope bias	$\delta \omega_{s2}^{b}=\left[\begin{array}{ccc} cos\omega_{1}t & \frac{cosat-cosbt}{2} & \frac{sinat+sinbt}{2}\\ 0 & cos\omega_{2}t & -sin\omega_{2}t\\ -sin\omega_{1}t & \frac{sinat-sinbt}{2} & \frac{cosat+cosbt}{2} \end{array}\right]\left[\begin{array}{c} \varepsilon_{x}^{s2}\\ \varepsilon_{y}^{s2}\\ \varepsilon_{z}^{s2} \end{array}\right]$	$\delta \omega_{is}^{b}=\left[\begin{array}{c} \varepsilon_{x}^{s}cos\omega t+\varepsilon_{y}^{s}sin\omega t\\ \varepsilon_{y}^{s}\\ -\varepsilon_{x}^{s}sin\omega t-\varepsilon_{y}^{s}cos\omega t \end{array}\right]$
Accelerometer bias	$\delta f_{s2}^{b}=\left[\begin{array}{ccc} cos\omega_{1}t & \frac{cosat-cosbt}{2} & \frac{sinat+sinbt}{2}\\ 0 & cos\omega_{2}t & -sin\omega_{2}t\\ -sin\omega_{1}t & \frac{sinat-sinbt}{2} & \frac{cosat+cosbt}{2} \end{array}\right]\left[\begin{array}{c} \nabla_{x}^{s2}\\ \nabla_{y}^{s2}\\ \nabla_{z}^{s2} \end{array}\right]$	$\delta f_{s}^{b}=\left[\begin{array}{c} \nabla_{x}^{s}cos\omega t+\nabla_{z}^{s}sin\omega t\\ \nabla_{y}^{s}\\ -\nabla_{x}^{s}sin\omega t+\nabla_{z}^{s}cos\omega t \end{array}\right]$
Modulation period	$T=\left(2\pi /\omega_{r1},2\pi /a,2\pi /\omega_{r2},2\pi /b\right)=6s$ ^1^	T=2π/ω=3s

^1^ $\omega_{r1}$ = ω = 120, $\omega_{r2}$ = 60, a = 180, b = 60.

**Table 4:** Parameters of the sensors in a simulation experiment.

Error Parameters	Gyroscope (X-, Y-, Z-Axis)	Accelerometer (X-, Y-, Z-Axis)
Bias	24°/h	2 mg
Random Walk	0.001°/$\sqrt{h}$	1000 ug/$\sqrt{Hz}$
correlated bias	0.001°/h	0.001

**Table 5:** Motion state.

Numerical Order	State	Time
1	Accelerate: 3 m/s2	1 s
2	uniform	19 s
3	Turnleft: 5°/s	5 s
4	uniform	15 s
5	Turnleft: 5°/s	5 s
6	uniform	15 s
7	Turnleft: 5°/s	5 s
8	uniform	15 s
9	Turnleft: 5°/s	5 s
10	uniform	15 s
11	Turnleft: 5°/s	5 s
12	uniform	15 s

**Table 6:** Maximum errors between two rotational modulation schemes comparison.

Maximum Errors	Single Axis RM Scheme	Compound RM Scheme
$\phi_{E}$ (″)	331.1649	24.0332
$\phi_{N}$ (″)	527.5398	29.1387
$\phi_{U}$ (′)	0.0316	0.0009
$\delta V_{E}$ (m/s)	1.5905	0.0408
$\delta V_{N}$ (m/s)	0.8949	0.0321
$\delta V_{U}$ (m/s)	0.0132	0.0008
$\delta P_{E}$ (m)	57.3105	0.4309
$\delta P_{N}$ (m)	109.6682	3.7567
$\delta P_{U}$ (m)	1.7212	2.3655

**Table 7:** Maximum errors between two rotational modulation schemes’ comparison.

Maximum Errors	Single Axis RM Scheme	Compound RM Scheme
$\phi_{E}$ (″)	11.6656	0.0961
$\phi_{N}$ (″)	2869.2883	0.1284
$\phi_{U}$ (′)	0.1253	0.0075
$\delta V_{E}$ (m/s)	8.2025	0.0652
$\delta V_{N}$ (m/s)	2.2987	0.0002
$\delta V_{U}$ (m/s)	0.0018	0.0005
$\delta P_{E}$ (m)	139.8875	1.1436
$\delta P_{N}$ (m)	396.7535	4.7562
$\delta P_{U}$ (m)	0.0642	0.0313

**Table 8:** Technical parameters of flight simulation turntable.

Rotation Rate Accuracy (°/s)	Rotation Rate (°/s)		
	Inner Frame	Middle Frame	Outer Frame
0.001	0.001–12,000	0.001–400	0.001–400

**Table 9:** Parameters of the sensors in experiment.

Error Parameters	Gyroscope (X-Axis)	Gyroscope (Y-, Z-Axis)	Accelerometer (X-Axis)	Accelerometer (Y-, Z-Axis)
Bias	30°/h	30°/h	5 mg	2 mg
Random Walk	0.30°/$\sqrt{h}$	0.30°/$\sqrt{h}$	150 ug/$\sqrt{Hz}$	90 ug/$\sqrt{Hz}$

**Table 10:** Maximum errors between two rotational modulation schemes comparison.

Maximum Errors	Single Axis RM Scheme	Compound RM Scheme
θ (°)	−6.25	2.98
γ (°)	−72.58	−0.80
ϕ (°)	13.78	5.36
$\delta V_{E}$ (m/s)	−579.63	15.71
$\delta V_{N}$ (m/s)	−88.29	−57.96
$\delta V_{U}$ (m/s)	−260.50	−1.386
$\delta P_{E}$ (m)	22,260	827.20
$\delta P_{N}$ (m)	−2926	−2558
$\delta P_{U}$ (m)	7268	−18.83

**Table 11:** Maximum errors between two rotational modulation schemes comparison.

Maximum Errors	Single Axis RM Scheme	Compound RM Scheme
(°)	−11	6.018
γ (°)	−65.3	0.8372
ϕ (°)	9.414	8.719

## Data Availability

Not applicable.
